# A Three-Gene Interferon Signature Predicts Sustained Complete Remission in Pediatric AML Patients

**DOI:** 10.3390/cancers18091423

**Published:** 2026-04-29

**Authors:** Shimaa Sherif, Aesha Ali, Khadega Ibrahim, Darawan Rinchai, Mohammed Elanbari, Dhanya Kizhakayil, Mohammed Toufiq, Fazulur R. Vempalli, Tommaso Mina, Patrizia Comoli, Kulsoom Ghias, Zehra Fadoo, Sheanna Herrera, Che-Ann Lachica, Enas D. K. Dawoud, Hani Bibawi, Sandra Sapia, Blessing Dason, Anila Ejaz, Mohammed Y. S. Anas, Ayman Saleh, Giusy Gentilcore, Davide Bedognetti, Chiara Cugno, Sara Deola

**Affiliations:** 1 Research Department, Sidra Medicine, Doha 26999, Qatar; ssherifkhedr@sidra.org (S.S.); aali11@sidra.org (A.A.); khadegaibrahim24@gmail.com (K.I.); drinchai@gmail.com (D.R.); medanbari@gmail.com (M.E.); dkizhakayil@sidra.org (D.K.); mohammed.toufiq@jax.org (M.T.); vrehaman@gmail.com (F.R.V.); sherrera@sidra.org (S.H.); clachica@sidra.org (C.-A.L.); edawoud1@sidra.org (E.D.K.D.); bdason@sidra.org (B.D.); ggentilcore@sidra.org (G.G.); davidebedognetti@gmail.com (D.B.); sdeola1@sidra.org (S.D.); 2 Pediatric Oncology and Hematology Department and Cell Factory, IRCCS Fondazione Policlinico San Matteo, 27100 Pavia, Italy; t.mina@smatteo.pv.it (T.M.); p.comoli@smatteo.pv.it (P.C.); 3 Department of Biological and Biomedical Sciences, Aga Khan University, Karachi 74800, Pakistan; kulsoom.ghias@aku.edu; 4 Department of Oncology, Aga Khan University, Karachi 74800, Pakistan; zehra.fadoo@aku.edu; 5 Pathology Department, Sidra Medicine, Doha 26999, Qatar; hbibawi@sidra.org (H.B.); ssapia-c@sidra.org (S.S.); 6 Weill Cornell Medicine–Qatar, Medical Education Division, Doha 24144, Qatar; 7 Pediatric Hematology-Oncology Division, Sidra Medicine, Doha 26999, Qatar; aejaz@sidra.org (A.E.); manas@sidra.org (M.Y.S.A.); asaleh@neomed.edu (A.S.); 8 Department of Internal Medicine, University of Genoa, 16132 Genoa, Italy

**Keywords:** tumor, AML, bone marrow microenvironment, interferon-related gene signature

## Abstract

The immunological composition of the microenvironment has shown relevance for diagnosis, prognosis, and therapy in solid tumors but remains underexplored in acute leukemias. We investigated the significance of the acute myeloid leukemia (AML) bone marrow microenvironment in predicting chemosensitivity and long-term remission in pediatric patients. We analyzed 32 non-promyelocytic pediatric AML patients at diagnosis using a NanoString PanCancer IO 360 assay, RNA sequencing, and deep-phenotype flow cytometry analyses. The findings were validated using the pediatric TARGET AML dataset. A short signature of three interferon (IFN)-related genes (*GBP1*, *PARP12*, and *TRAT1*) distinguished patients with chemosensitive disease and reduced minimal residual disease after induction chemotherapy. The signature stratified patients overall, and within the clinically defined “standard-risk” group, patients with high gene expression at diagnosis had significantly longer overall survival. The leukemia microenvironment associated with this signature showed enrichment of non-exhausted CD4^+^ and CD8^+^ T cytotoxic lymphocytes and expansion of CD8^+^ T effector memory cells re-expressing CD45RA (TEMRA) in patients with a favorable prognosis. Our results show the importance of the bone marrow microenvironment in pediatric AML and provide tools for a refined stratification of “standard-risk” patients, lacking adequate risk-oriented therapies. They also offer a promising guide for tackling immune pathways and exploiting immune-targeted therapies.

## Simple Summary

Acute myeloid leukemia in children is a challenging disease, and it is difficult to predict which patients will respond well to treatment. Most current therapies focus on leukemia cells, but growing evidence suggests that the surrounding bone marrow environment, especially the immune system, may play an important role. In this study, we examined whether immune-related patterns that exist at diagnosis could help to recognize children who are more likely to respond to chemotherapy and achieve long-term remission. We discovered three genes linked to immune activity that are associated with better treatment response and more active, effective immune cells. These findings reveal the importance of the bone marrow environment and suggest that incorporating immune information could improve patient risk assessment and support the development of more personalized and immune-based treatment strategies.

## 1. Introduction

Pediatric acute myeloid leukemia (AML) represents a complex and heterogeneous group of hematological malignancies that account for 20% of all pediatric leukemias. Despite remarkable breakthroughs in therapeutic approaches in the last decades, AML is still characterized by suboptimal outcomes, with an overall survival (OS) rate of approximately 65% [1,2]. Recent improvements in disease-free survival and risk of relapse have been achieved by the addition of a targeted tyrosine kinase inhibitor in patients with FLT3/ITD+ [3].

Numerous studies have emphasized the critical role of immune cell composition and behavior in the tumor microenvironment (TME) of solid cancers [4,5]. The contexture, functional orientation, and intricate interactions of immune cell subsets and tumor cells within the TME can directly influence patients’ treatment response and clinical outcomes [6,7,8].

Although it is tempting to speculate that the same model may be applicable to acute leukemia to exploit novel immunotherapies and extend patient survival [9,10,11], the role of TME in leukemia (L-TME) is still underexplored [12]. The architecture of the bone marrow (BM) sustains normal hematopoiesis through delicate interactions in microenvironment subcompartments [13]. Within BM niches, resident immune cells such as antigen-presenting cells, B cells, and memory T cells allow cognate antigen interactions and exchange immune information with the peripheral blood (PB) [14]. At the onset of leukemia, the homeostasis of these fine-tuned networks is disrupted by the rapid invasion of leukemic cells, leaving a limited healthy counterpart available for assessment.

To increase the likelihood of meaningful immune network analysis in this scenario, we performed a gene expression study starting from an immune-directed platform, the NanoString PanCancer immune-oncology assay.

We explored the importance of the leukemic BM microenvironment (L-TME) in predicting chemosensitivity and ≥6 months remission in a multi-center group of 32 non-promyelocytic pediatric AML patients and validated the findings using data from the Therapeutically Applicable Research to Generate Effective Treatments (TARGET) AML database. Finally, we confirmed our results using 20-color flow cytometry analysis of the L-TME lymphoid composition on all available live-frozen BM samples in the cohort (nine samples).

## 2. Materials and Methods

### 2.1. Ethical Approval

The study was conducted in accordance with the ethical approval granted at all collaborative sites: Qatar, Sidra Medicine Institutional Review Board (IRB: #1804022328; Pakistan, Aga Khan University Ethics Review Committee# 3825-Onc-ERC-15; Italy, IRCCS Fondazione Policlinico San Matteo Ethics Review Committee #1500786 and 1500787). Informed consent for participation in the study was obtained, and all samples were collected after obtaining the informed consent as appropriate.

### 2.2. Clinical Samples and Cohorts

Thirty-two pediatric patients recruited in Qatar, Italy, and Pakistan provided BM samples for AML diagnosis. The patients were stratified and treated according to the COG AAML0531 protocol [15]. Samples were live-frozen after Ficoll-gradient enrichment and channeled to Sidra Medicine for downstream analyses. Clinical and follow-up data for each patient were recorded, as detailed in Supplementary File S1.

Of the 32 patients, 25 were analyzed using NanoString (Seattle, WA, USA; RRID:SCR_023912) and formed the discovery cohort. A subset of 19 patients from the discovery cohort, from whom sufficient RNA was available, was subsequently studied using mRNA-Seq for technical validation (cross-validation subset). Next, an independent small cohort of seven newcomer patients was assessed using RNA-Seq, and an internal validation cohort was formed. For pathway and gene enrichment analyses, all RNA-Seq cohorts (RNA cross-validation subset, *n* = 19, and internal validation cohort, *n* = 7) were combined in the “whole-RNA-Seq” cohort (Supplementary File S1).

Patients achieving a 6-month complete remission (CR) after the first line of chemotherapy were assigned to the “sustained-CR” group, while patients who were refractory, died, or relapsed within 6 months and/or obtained CR after allogeneic hematopoietic stem cell transplantation (allo-HSCT) were assigned to the “non-sustained CR” group.

Five patients from the “non-sustained CR” group and four patients from the “sustained-CR” group were analyzed using a 20-color panel by flow cytometry (deep phenotype cohort).

The Therapeutically Applicable Research to Generate Effective Treatments (TARGET) pediatric AML RNA-Seq database AAML1031 [3] was used as the external validation cohort (RRID:SCR_014514). Patients with BM samples at diagnosis and complete clinical data available (*n* = 833) were selected and assigned to “sustained-“ and “non-sustained CR” groups with the same criteria applied to the cohort of 32 patients (Supplementary File S2). To harmonize the risk stratification category nomenclature across different protocols, we use in the manuscript “standard” risk, referring to standard- or intermediate-risk patients.

### 2.3. RNA Analysis

BM samples were analyzed for RNA expression using the nCounter NanoString PanCancer immune-oncology IO 360 assay (Seattle, WA, USA; RRID:SCR_021712), which consists of a 770-gene panel enriched for immune-related genes. mRNA-sequencing (mRNA-seq) was performed at a depth of 20 million reads on an Illumina HiSeq 4000 sequencer (Illumina, San Diego, CA, USA; RRID:SCR_016386). Data analysis from fastq to the raw count generation was performed using the bcbio-nextgen v1.2.0 (RRID:SCR_004316) rnaseq pipeline. The preliminary quality of sequencing reads was assessed using FASTQC v.0.11.8 (RRID:SCR_014583). Raw reads were mapped to the human genome GRCh38.p13 (Genome Reference Consortium Human Build 38, INSDC Assembly GCA_000001405.28, Dec 2013) using STAR_2.6.1d aligner (RRID:SCR_004463), and featureCounts v2.0.0 (RRID:SCR_012919) was used to generate the raw counts.

Gene expression normalization for all cohorts was performed using the variance stabilizing transformation (VST) of the DESeq2 R package (v(1.44.0); RRID:SCR_015687) within lanes to correct for gene-specific effects (including GC content) and between lanes to correct for sample-related differences (including sequencing depth) using the EDASeq v. 2.30.0 R package (RRID:SCR_006751).

### 2.4. Flow Cytometry Analysis

Patients with available live frozen BM cells (sustained CR, *n* = 4; non-sustained CR, *n* = 5, as described in Supplementary File S1) were analyzed using a 20-color flow cytometry panel to characterize the infiltration of T, B, and NK cells in the L-TME. The cell number and viability were determined using a NucleoCounter^®^ NC-200™ (ChemoMetec, Allerod, Denmark; RRID:SCR_025290). Cells were stained with Zombie UV Fixable Viability Dye (BioLegend, San Diego, CA, USA, Cat#423108), treated with human Fc receptor block (BD Pharmingen, San Diego, CA, USA, Cat# 564219; RRID: AB_2728082) and mouse serum (Invitrogen, Carlsbad, CA, USA, Cat#01-6501), stained according to the manufacturer’s instructions with antibody cocktails (Supplementary File S3) extracellularly and intracellularly (FIX & PERM Cell Kit, Invitrogen, Carlsbad, CA, USA, Invitrogen Cat# GAS003), and acquired on a FACSymphony A5 (BD Biosciences, San Jose, CA, USA; RRID:SCR_022538). Data analysis was conducted using the OMIQ software (Dotmatics, Boston, MA, USA: www.omiq.ai, www.dotmatics.com).

### 2.5. Statistical Analysis

All analyses, unless otherwise specified, were performed in R v.4.2.1 (RRID SCR_001905). DEGs were analyzed using a log2 normalized expression matrix using limma v. 3.52.4 (RRID:SCR_010943) [16] unadjusted *p* < 0.05 and visualized with ggplot2 v. 3.4.2 (RRID:SCR_014601) [17] and ComplexHeatmap v. 2.12.1 (RRID:SCR_017270) [18].

Survival analysis was conducted using Survminer v.0.4.9 (RRID:SCR_021094) [19] to generate Kaplan–Meier curves. Hazard ratios (HRs) and corresponding *p*-values were calculated by Cox proportional hazard regression analysis using the R survival package v.3.5.7 (RRID:SCR_021137) [20]. The overall *p*-value for comparing survival among the groups was determined using the log-rank test. Clinical variables included age at diagnosis (ordinal), clinical risk groups (categorical) and three-gene signature enrichment divided into quartiles (categorical).

For pathway enrichment analysis, the DEGs in the CR groups (*p* < 0.05) from the whole-RNA-Seq combined cohort (19 + 7 samples) were uploaded to Ingenuity Pathways Analysis (IPA, QIAGEN, Redwood City, CA, USA; RRID:SCR_008653). Raw data were downloaded into R and plotted using the R package ggplot2 (v. 3.4.2).

Single-sample-gene-set enrichment Analysis (ssGSEA) was performed using normalized log2 expression data to calculate gene enrichment scores (ESs) using the R package GSVA v. 1.44.5 (RRID:SCR_021058) [21]. Gene sets of immune cell-specific signatures were used as described by Bindea et al. [22] with slight modifications [23]. ESs were visualized using the ComplexHeatmap R package (v. 2.12.1) [19]. Correlations were analyzed using Stat_cor in the ggpubr R package v. 0.6.0 (RRID:SCR_021139). Statistical analyses of the flow cytometry data were performed using GraphPad Prism v. 10.3.1 (RRID:SCR_002798).

## 3. Results

### 3.1. Patients with Sustained CR Present a TH1-Enriched L-TME at AML Diagnosis

**Figure 1:**
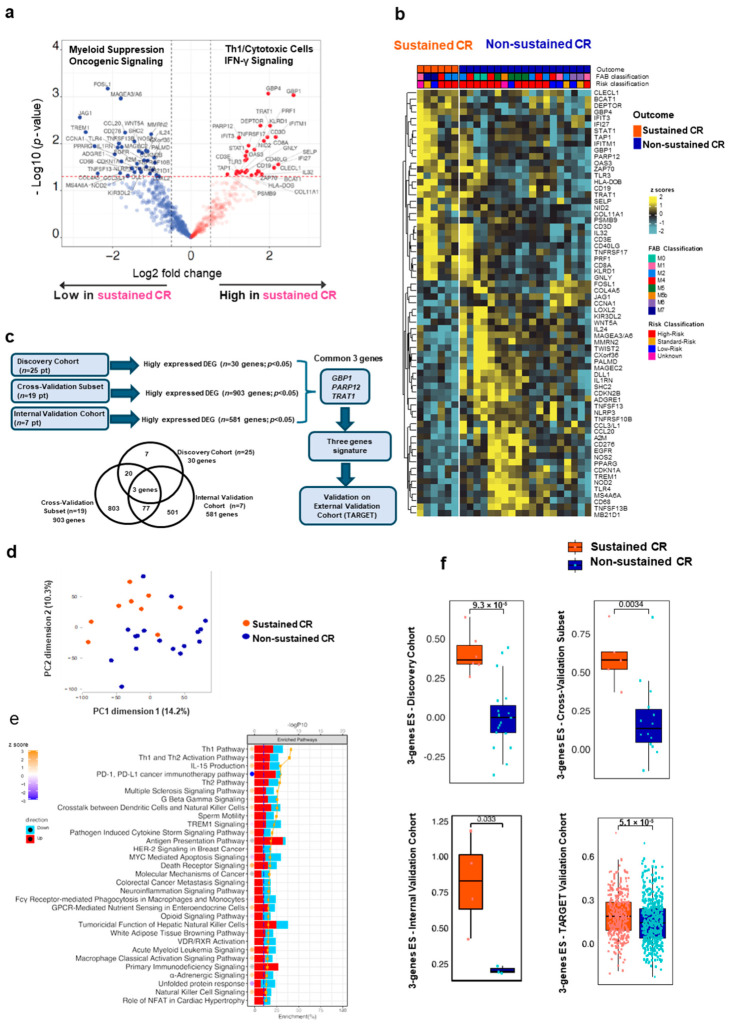
Identification of prognostic signature from the gene expression data of the discovery cohort, cross-validation subset, and internal validation cohort. (**a**) Volcano plot of differentially expressed genes in sustained CR vs. non-sustained CR from the discovery cohort (*n* = 25). Red dots, high-expressed genes in the CR sustained groups (*p*-value  <  0.05, Log2 fold change > 0); blue dots, low-expressed genes in the CR sustained groups (*p*-value  <  0.05, Log2 fold change < 0). This statistical threshold was consistent across the discovery, cross-validation, and validation cohorts. (**b**) Heatmap showing the expression of 67 DEGs between the sustained CR and non-sustained CR from the discovery cohort (*n* = 25). FAB classification and clinical risk stratification are annotated on top of the heatmap. (**c**) A chart and Venn diagram showing the overlap among the highly expressed (upregulated DEG in sustained CR) genes from the discovery cohort (*n* = 25), the cross-validation subset (*n* = 19), and the internal validation cohort (*n* = 7). The intersection of these cohorts resulted in three common genes (*GBP1*, *PARP12*, and *TRAT1*). The selection was performed by shortlisting significant DEGs (*p* < 0.05) overexpressed in the sustained-CR group across all the cohorts. (**d**) PCA plot of the normalized expression values of the 26 patients from the whole mRNA-Seq cohort annotated by CR outcome. (**e**) IPA pathways analysis of DEGs (*p* < 0.05) between the sustained CR and non-sustained CR from whole mRNA-Seq cohort (*n* = 26). Red, upregulated in sustained CR; blue, downregulated in sustained CR. The orange line indicates the −log (*p*-value). The dots display the predicted activation state of the implicated biological functions reflected by the activation z score. The bases of this inferred activation state are literature-derived relationships between genes and the corresponding biological function. Pathways that are activated are marked with orange dots, indicating a positive activation score, whereas pathways inhibited are marked with a blue dot, indicating a negative activation score. A gray circle indicates that no literature-derived information exists to estimate the activation state. (**f**) Boxplots displaying the enrichment scores (ES) for the three-gene signature between the sustained CR and non-sustained CR in the different cohorts. The *p*-value is calculated by Student’s *t*-test, and the middle black line represents the median.

We performed DEG analysis using the NanoString assay on the discovery cohort between patients with sustained CR (*n* = 6) vs. non-sustained CR (*n* = 19). Due to the limited number of patients in this cohort, we relaxed the stringency of the statistical analysis, choosing to filter all values with *p* ≤ 0.05 instead of applying FDR correction. We identified a clear distinction between myeloid suppression-related genes and a T-cell infiltration signature represented by 67 DEGs (*p* ≤ 0.05) (Figure 1a) (Supplementary File S4). By mapping these genes in the two CR groups, the T-cell signature, including cytotoxic T-cell infiltration (*CD3D*, *CD8A*, *PRF1*, and *GNLY*), interferon (IFN) signaling activation (*IFIT3*, *IFITM1*, *IFI27*, *STAT1*, *GBP1*, *PARP12*, and *TRAT1*), antigen presentation, and B-cell functions (*TAP1* and *CD19*), characterized patients with sustained CR, while early loss of CR and refractory disease were marked by a pro-inflammatory response of the macrophage-myeloid suppression signature (*CD68* and *TREM1*) and an intrinsic oncogene signaling linked to T-cell exclusion and immune suppression (*WNT5A*, *WNT-β*-catenin pathway, and *EFGR*) (Figure 1b).

To validate these results, we performed DEG analysis using mRNA-Seq in a subset of 19 patients from the discovery cohort (cross-validation subset), obtaining a total of 903 DEGs overexpressed in the sustained CR cohort, overlapping with the previous analysis. We then replicated the analysis in the internal validation cohort, obtaining 581 overexpressed genes in the sustained CR cohort (Figure 1c; DEG lists are available in Supplementary Files S5 and S6). Finally, we combined the mRNA-Seq-based cohorts (whole mRNA-Seq cohort; see Supplementary File S1) to perform functional pathway analyses on the DEGs in patients with sustained- vs. non-sustained-CR patients (Supplementary File S7). As for the discovery cohort, we used a *p* ≤ 0.05 filter for both the cross-validation subset and the internal validation cohort, rather than applying FDR correction. This approach was chosen again to ensure that a wider set of genes could pass selection criteria in such limited patient size groups.

By applying principal component analysis (PCA) to the transcriptome of the whole-mRNA-Seq cohort, we observed a separation pattern between sustained and non-sustained CR (Figure 1d), suggesting biological differences between the compared groups. IPA enrichment pathway analysis confirmed that patients with sustained CR benefit from an immune-rich L-TME, with a prevalence of T helper 1 (TH1) activation pathways, IL-15 production, and inhibition of PD1–PDL1 and myeloid TREM1 pathways, demonstrating an association between a TH1-skewed/cytotoxic L-TME and long-term remission (Figure 1e, Supplementary File S8).

### 3.2. A TH1-Enriched L-TME at AML Diagnosis Is Represented by a Three-Gene IFN Signature

To restrict this gene signature to a robust and clinically applicable one, we shortlisted the genes that were (1) common across all cohorts and (2) most highly expressed in DEG analysis. We identified three genes: guanylate-binding protein (*GBP1*), poly-ADP-ribose-polymerase-12 enzyme (*PARP12*), and T-cell receptor-associated transmembrane adapter (*TRAT1*) (Figure 1c). Notably, all three genes are related to IFN: *GBP1* [24,25] and *PARP12* [26] are type II and type I IFN-stimulated genes’ innate immunity enhancers with strong antimicrobial properties, while *TRAT1* is involved in cytotoxic/IFNg-polarized immune responses [27] and correlates with immune cell infiltration and favorable prognosis in non-small-cell lung carcinomas and diffuse large B-cell lymphoma [27,28].

To demonstrate the stability of the three-gene combination across the cohorts, we further analyzed the gene set with a “leave-one-out” validation (LOOV) and a “bootstrap” resampling analysis. *GBP1* and *PARP12* demonstrated a very good stability across all cohorts, while *TRAT1* was well conserved in the discovery cohort but less stable in the cross-validation and internal validation cohorts (Supplementary Figure S1a; see details about the types of tests in the figure caption).

Finally, we validated the identified IFN signature in the TARGET database to evaluate its potential clinical relevance.

In the TARGET cohort, the three-gene IFN signature was significantly enriched in patients with sustained CR (Figure 1f) and performed better than all other genes in identifying patients with the lowest hazard ratio (HR) (Supplementary Figure S1b).

### 3.3. The L-TME of Patients with the Three-Gene IFN Signature Is Enriched of Non-Exhausted CD4^+^ and CD8^+^ T Cytotoxic Lymphocytes

**Figure 2:**
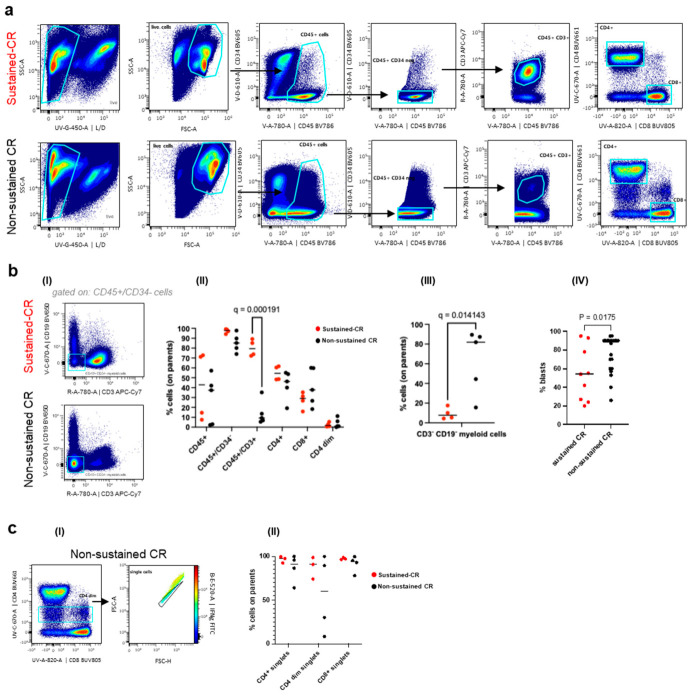
Lymphocyte profiling of the L-TME in pediatric AML patients. (**a**) Logical gating strategy showing the T cell composition of L-TME in AML patients with (top row, *n* = 4) and without (bottom row, *n* = 5) sustained CR. The data are displayed cumulatively in the dot plots and individually in the graphs (**b**,**c**). The same cumulative and individual representation is depicted in all figures showing flow cytometry data. (**b**) (**I**) Cumulative plots displaying CD45^+^/CD34^−^/CD19^−^ and CD3^−^ myeloid cells in AML patients with (top row, *n* = 4) and without (bottom row, *n* = 5) sustained CR. (**II**,**III**) Individual values of samples in (**a**,**b**) are displayed in a graph; the Q value is the result of FDR-corrected multiple *t*-tests between sustained (red) and non-sustained (black) patients’ samples. Only significant differences are shown. (**IV**) The percent of blasts scored in the patients’ diagnosis for all the cohort is displayed in the graph for patients with and without sustained CR. The blast score was calculated by a combination of microscope and clinical flow cytometry analyses and provided as clinical report for patients in the Pathology Laboratory of Sidra. (**c**) (**I**) Phenotype of the CD4^dim^ cell population: the majority of the cells are IFNγ^+^ (shown in the colored *Z*-axis) CD4^+^ and CD8^+^ doublets, engaging in close networking interactions. A full panel of expression markers for this population is shown in Supplementary Figure S1c. (**II**) Individual values of samples in (**b**) show a trend of enrichment in CD4^dim^ doublets (considering lower singlets) in patients without long-term remission.

To explore the biological context behind our findings, we analyzed the L-TME of all available live-cryopreserved BM samples (*n* = 9; 5 non-sustained CR and 4 sustained CR) in our cohort using a 20-color lymphoid-skewed antibody panel (Supplementary File S3). The number of patients with available cells for analyses was very limited; therefore, all the results are to be considered preliminary. The CD45^+^ healthy L-TME portion was comparable between sustained- and non-sustained-CR samples (97.2 ± 2% vs. 85.4 ± 9.1%, respectively). However, the cellular composition was strongly skewed, with a higher frequency of CD3^+^ T-lymphocytes in the sustained-CR (80.1 ± 8.3%) compared with the non-sustained-CR group (14.1 ± 12.2%) (Figure 2a,(bI,cI)) and a decrease in the myeloid healthy CD3^−^/CD19^−^ compartment (9.4 ± 6% in sustained CR and 63.7 ± 32.5% in non-sustained CR (Figure 2(bI,bIII)). A healthy age-matched BM to our cohort typically contains 50–70% myeloid cells, while lymphocyte levels range from 10 to 30% [29], suggesting that lymphocytes in the sustained-CR cohort actively expanded in the presence of leukemia blasts at the expense of the myeloid cell population. The percent of blasts reported at the time of clinical AML diagnosis (combined microscope and flow cytometry analyses) was also lower in the sustained-CR cohort (Supplementary File S1, Figure 2(bIV)). A deeper analysis of the T lymphocyte subpopulations revealed that the frequencies of CD8^+^ and CD4^+^ cells were comparable between the two groups (Figure 2a,(bII)). Curiously, a small population of CD4^dim^/CD8^+^ or ^−^ cells characterized more non-sustained remission samples (Figure 2(cI,cII)), representing IFNg^+^ doublet T-cells engaged in close contact with other CD8^+^ and CD4^+^ cells, likely in cytolytic activity (see also Supplementary Figure S1c).

**Figure 3:**
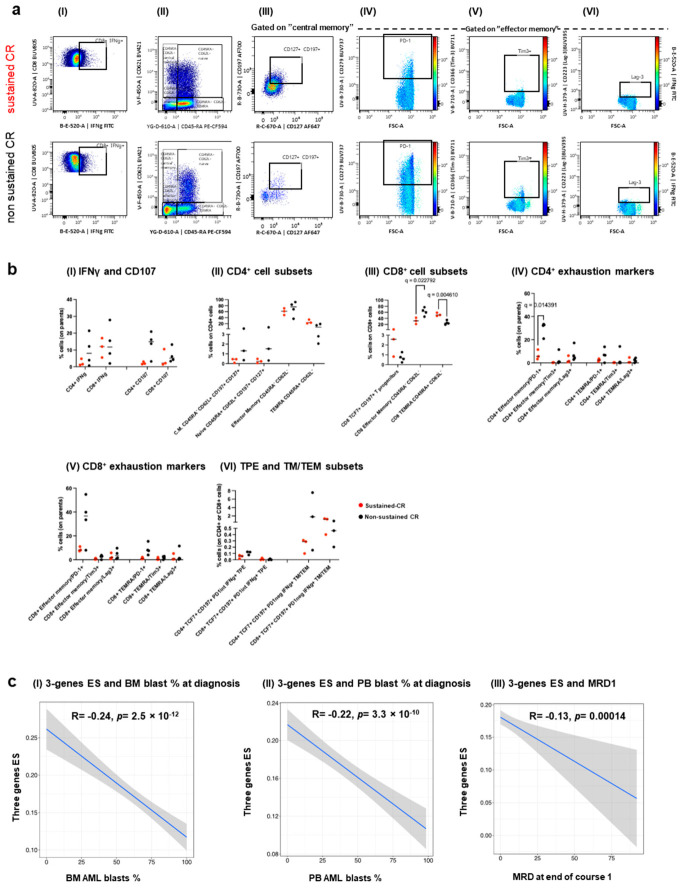
Characterization of lymphocyte subsets in the L-TME of pediatric AML patients. (**a**) Logical gating strategy, showing the measurement parameters of CD8^+^ cells (same logical gating was used for CD4^+^ cells): (**I**) IFNγ positive cells; (**II**) “central memory”, “naïve”, “effectors”, and “TEMRA” cells; (**III**) gating strategy to calculate naïve and central memory cells; and (**IV**–**VI**) PD-1, TIM-3, and LAG-3 exhaustion markers gated on “effector memory” cells in sustained- and non-sustained-CR patients. The data are displayed cumulatively. Plots in (**IV**–**VI**) show IFNγ in the colored *Z*-axis. (**b**) (**I**–**V**) Individual values of samples, measured with the gating strategy in (**a**), are displayed in graphs. Some samples were excluded from the statistical analyses of subpopulations, due to an insufficient number of acquired events; in these analyses we included (*n* = 3) for sustained-CR and (*n* = 3–4) for non-sustained CR patients. Naïve and central memory cells were measured with the gating strategy shown in Figure 3(aII,aIII) selecting CD127^+^ (IL7Ra) and CD197^+^ (CCR7) on cells gated in (**II**). (**VI**) T progenitor exhausted (TPE) and T non-exhausted (TM/TEM) cells were measured according to criteria published in [30]. Further gating strategies are shown in Supplementary Figures S2 and S3b. The Q value is the result of FDR-corrected multiple *t*-tests between sustained (red) and non-sustained (black) patients’ samples. Only significant differences are shown. (**c**) Spearman correlations showing the relationship between the three-gene ES score and the % of AML (**I**) BM and (**II**) PB blasts at AML diagnosis and (**III**) with the burden of MRD measured after the first induction therapy (MRD1) in the TARGET cohort. The blue line displayed in the figure represents a linear regression fit line. The shaded area surrounding the line represents the 95% confidence interval of the linear regression estimate.

The relative expression of IFNg and CD107 was similar between the two cohorts, trending higher in non-sustained-CR CD4^+^ cells (Figure 3(aI,bI)); however, the cell composition between the cohorts showed differences. First, CD8 T effector memory cells (CD8^+^/CD62L^−^/CD45RA^−^ TEM) were enriched in the non-sustained remission cohort, whereas CD8 T effector memory cells re-expressing CD45RA (CD8^+^/CD62L^−^/CD45RA^+^; TEMRA) were more prevalent in the sustained remission cohort (Figure 3(aII,bII,bIII)). Second, TEM cells in the non-sustained remission cohort showed a larger FSC and a more exhausted phenotype, with PD-1 expression being higher in the CD4^+^ compartment. Other exhaustion markers (TIM-3 and LAG-3) also trended toward higher levels than those in the sustained remission group (Figure 3(aIV–VI,bIV,bV)).

We then assessed the L-TME T-cell memory compartment, according to a paired phenotype/TCR-functional classification recently described by Oliveira et al. in the context of melanoma [30]. The paper described intra-tumor infiltrating memory T cells as (1) primarily TCR-tumor specific but exhausted (T progenitor exhausted (TPE): TCF7^+^/CCR7^+^/PD-1^low/Int^/absent cytotoxic potential) and (2) viral/other non-primarily tumor TCR specific non-exhausted (T memory/T effector memory (TM/TEM): TCF7^+^/CCR7^+^/PD-1^−^/high effector cytokines). The frequencies of TPE and TM/TEM were similar in both groups, with a trend toward a higher presence in the non-sustained remission group (Figure 3(bVI) and Supplementary Figure S2). Finally, the presence of both CD4^+^ and CD8^+^ naïve and central memory cells, measured as CD62L^+^/CD45RA^+^/CD197^+^/CD127^+^ and CD62L^+^/CD45RA^−^/CD197^+^/CD127^+^, respectively; CD19^+^ B cells; (CD8^+^/CD3^−^/CD56^+^) NK; and (CD8^+^/CD3^+^/CD56^+^) NKT cells was minimal and similar in both groups (Figure 3(aII,aIII,bII), and Supplementary Figure S3a–c).

Collectively, the phenotypic data suggested functional differences in the L-TME of patients with different prognoses, showing a limited and more exhausted T-cell compartment in the L-TME of non-sustained-CR patients and a bulky-expanded less-exhausted T-cell compartment in patients with sustained CR.

In this context, it is likely that our three-gene IFN-related RNA signature captures a large abundance of functionally active T cells, approximately six folds higher (80% vs. 14% CD3^+^ infiltration) in patients with sustained remission.

### 3.4. A High Three-Gene IFN Signature Enrichment at AML Diagnosis Is Inversely Proportional to AML Leukemic Burden

The lower number of blasts observed in the sustained-CR cohort suggests that a functionally T-cell-enriched L-TME could contribute up front (at AML onset) to contain the AML blast burden.

To test this hypothesis, we verified the relationship between the number of blasts in the BM and PB at AML diagnosis and the three-gene IFN score in the TARGET cohort.

Both BM and PB blasts at diagnosis showed a clear inverse correlation with three-gene ES (Figure 3(cI,cII)). Although BM and PB blast numbers are not considered prognostic factors in pediatric non-promyelocytic AMLs (we confirmed no OS correlation with these variables in the TARGET cohort), minimal residual disease after the first induction cycle (MRD1) is a clear biomarker of disease clearance and was found to have prognostic significance in the most recent WHO (adults) and COG AAML1831 (pediatric) classifications [31]. We tested this variable in the ES and found an inverse correlation between the enrichment of the three-gene signature and the burden of MRD1 (Figure 3(cIII)).

These findings strengthen the evidence that a high three-gene ES represents a functional immune milieu at AML onset, contributing to disease eradication.

### 3.5. A High Three-Gene IFN Signature Enrichment at AML Diagnosis Confers a Longer OS to AML Standard-Risk Patients

In COG AMML 0531 and 1031 protocols, the intensity of the treatment received by AML patients is calibrated on the relapse risk categorized as “low,” “standard”, and “high” based on cytogenetic abnormalities and early response to treatment [15]. The discovery and validation (TARGET) cohorts were then stratified accordingly. Refined classifications integrating novel genomic data, including MRD1, are currently available for AML patients [31,32]. However, standard-risk patients are in the “gray zone” of a category formed by exclusion criteria from high- and low-risk definitions. An allo-HSCT intensified treatment is usually offered if an HLA-matched related donor is available [15,33].

**Figure 4:**
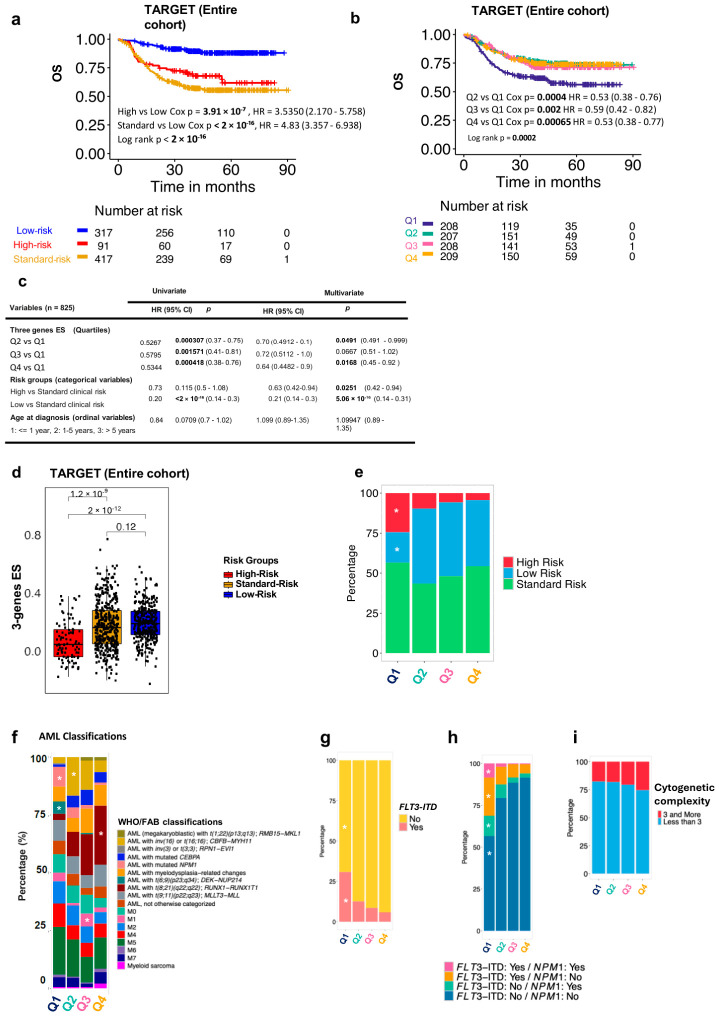
Prognostic value of the three-gene IFN signature in pediatric AML OS. (**a**) Kaplan–Meier OS curves for clinical risk groups in the TARGET cohort: low-, standard-, and high-risk groups. (**b**) Kaplan–Meier OS curve for the three-gene enrichment score (ES) groups: The enrichment score was split into four quartiles within the entire TARGET cohort, with Q1 representing the lowest score and Q4 being the highest. (**c**) Univariate and multivariate analyses of OS in the TARGET cohort, evaluating the three-gene ES quartiles, clinical risk-stratified groups, and age at diagnosis (*n* = 825). (**d**) Boxplots showing the three-gene ES across the clinical risk groups. *p*-values were calculated using the Student’s *t*-test, and the central black line represents the median. (**e**) Stacked bar chart showing the distribution of clinical risk groups across the quartiles of the ES signature in the TARGET cohort. White asterisks indicate *p*-value < 0.05, based on a chi-squared test for equal proportions (*n* =789). (**f**) Distribution of AML clinical subsets according to WHO and FAB classifications across the different three-gene ES quartiles in the TARGET cohort. White asterisks indicate *p*-value < 0.05, based on a chi-squared test for equal proportions (*n* = 784). A higher proportion of patients with AML *t(8;21)(q22;q22)*; *RUNX1-RUNX1T1* was observed in the highest three-gene ES quartile (Q4) than in the other ES groups (*p* = 7.83 × 10^−10^). A higher proportion of patients with AML with mutated *NPM1* and *t(6;9)(p23;q34)*; *DEK-NUP214* was present in the lowest three-gene ES quartile (Q1) compared with other ES groups (*p* = 0.00031 and 4.282 × 10^−6^, respectively). (**g**,**h**) Stacked bar charts showing (**g**) the distribution of *Flt3-ITD* mutations across the ES quartiles and (**h**) the distribution of combined *Flt3-ITD* and *NPM1* mutations. The white asterisk indicates a *p*-value of 4.685 × 10^−13^ based on a chi-square test for equal proportions of *FLT3-ITD* mutation status across ES quartiles (*n* = 796) and a *p*-value < 2.2 × 10^−16^ for combined *Flt3-ITD* and *NPM1* mutations status across score quartiles (*n* = 789). (**i**) Stacked bar chart showing the distribution of cytogenetic complexity groups within the quartiles of the ES signature in the TARGET cohort (*n* = 789). Patients with <3 cytogenetic alterations are shown in blue, and those with ≥3 cytogenetic alterations are shown in red.

In the TARGET cohort, the OS of standard-risk patients’ OS is significantly lower than that of low-risk patients and comparable with that of the high-risk group that benefits from an intensified treatment (Figure 4a).

We performed a long-term (90-months) OS analysis of the TARGET cohort according to the three-IFN gene signature, quantified in “quartiles” enrichments, and we discovered that the lowest ES “Q1” in the L-TME of patients was associated with a significantly lower OS (Figure 4b). To benchmark the importance of ES against clinically relevant parameters (cytogenetics, molecular mutations, and morphological remission after first induction), we performed univariate and multivariate analyses measuring the ES quartiles against the clinical-risk group stratification, which included all the relevant variables. The importance of the ES in determining the OS was maintained particularly in the extreme enrichments Q1 (lowest) vs. Q4 (highest) in the multivariate analysis (Q4 vs. Q1, *p* = 0.0168; Figure 4c).

Next, we analyzed the distribution of the three-IFN gene signature across clinical risk groups. The signature was enriched in the low- and standard-clinical risk groups (Figure 4d). Accordingly, high-risk patients were enriched and low-risk patients were depleted of the less favorable Q1 ES signature, whereas the quartiles were evenly distributed across standard-risk patients (Figure 4e).

When we analyzed the scores across the FAB and WHO AML classifications, we found that patients with low-risk *t(8;21)(q22;q22.1)/RUNX1:RUNX1T1* translocations were predominantly represented in the Q4 (high ES) group, whereas those with *NPM1* mutations and *t(6:9)(p23;q34)/DEK:NUP214* translocations were more prevalent in the unfavorable Q1 (low ES) group (Figure 4f). Next, we screened the distribution of scores of patients with *Flt3-ITD* mutations. An unfavorable Q1 quartile was more prevalent among *Flt3-ITD* mutated patients (Figure 4g). When *Flt3-ITD* was combined with *NPM1* mutation, Q1 remained predominant across all *Flt3-ITD/NPM1* combinations, whereas Q2-4 were enriched in patients without mutations (Figure 4h). Finally, we checked the quartile distribution in patients with or without complex cytogenetics (≥3) and found that the quartiles were evenly distributed in these patients (Figure 4i).

**Figure 5:**
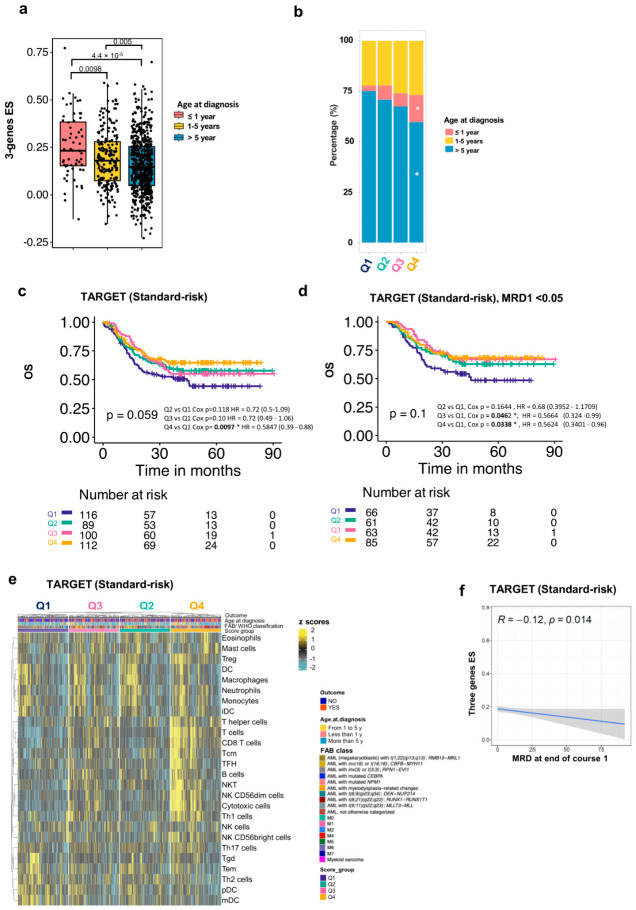
Three-gene IFN signature and OS in standard-risk pediatric AML patients. (**a**) Boxplots showing the three-gene ES across different age groups at diagnosis. *p*-value was calculated by Student’s *t*-test, and the central black line within each box represents the median. (**b**) Stacked bar chart showing the distribution of the variable “age at diagnosis” within the quartiles of the ES signature in the TARGET cohort. The chi-square pairwise proportion test showed the lowest proportion of the age group >5 years in patients with the highest three-gene ES compared with other ES groups (*p* = 0.0058) and a higher proportion of age group ≤1 year in the same group (*p* = 0.00054). White asterisks indicate *p*-value < 0.05, based on a chi-squared test for equal proportions. (**c**) Kaplan–Meier OS curve for the three-gene ES groups split in quartiles in the clinical standard-risk group of the whole TARGET cohort. *p*-values of the compared quartiles, obtained with a Cox regression analysis, are annotated in the graph (*n* = 417). (**d**) Kaplan–Meier OS curve for the three-gene ES groups split in quartiles in the clinical standard-risk group of the TARGET cohort after removing all patients with MRD1 ≥ 0.05. *p*-values from Cox regression analyses comparing quartiles are annotated in the graph (*n* = 275). (**e**) Heatmap displaying immune cell subpopulation enrichment scores across three-gene ES groups within the standard clinical risk patients’ group. These immune signatures were previously described in Sherif et al. [23]. For each ES group CR outcome AML classification, outcome (sustained/non-sustained CR) and age at diagnosis are annotated on top of the heatmap. (**f**) Spearman correlations showing the relationship between the three-gene ES score and the burden of MRD measured after MRD1 in the clinical standard-risk group. The blue line displayed in the figure represents a linear regression fit line. The shaded area surrounding the line represents the 95% confidence interval of the linear regression estimate.

Surprisingly, the ES was higher in infants (Figure 5a,b). A negative correlation between age and three-gene ES was further confirmed by correlation analysis (Supplementary Figure S3d). This finding could not be assessed in our cohort, as only one patient was younger than 1 year old. Still, it aligns with the recent literature suggesting that infant T cells may have innate-like functions and are prompt to respond to danger signals response [34,35,36]. Indeed, the possibility of stratifying infant AML using a favorable prognostic factor could be promising, considering that this patient group usually has poor clinical outcomes [37].

To investigate whether the three-gene score could improve stratification within the risk groups, we tested the OS in each group. A survival advantage was granted by high three-gene ES only in the standard-risk cohort (Q1 vs. Q4, *p* = 0.0097) (Figure 5c).

Specifically, a logistic regression analysis revealed that children with standard-risk AML and in the Q4 high three-gene ES quartile had 100% increased odds of achieving long-term OS compared with those in the Q1 low ES quartile (Supplementary Figure S3e).

While we lacked sufficient genetic information to re-stratify patients according to the latest COG AAML1831 protocol and test the three-gene ES in these new risk categories (33), we used MRD1 data to assign MRD1 ≥ 0.05% to high risk following the newest recommendations. While the OS in all TARGET cohorts did not substantially change (Supplementary Figure S3f), the three-gene ES in the standard-risk cohort still highlighted a significant negative impact on OS for patients with a low score (Q1) (Figure 5d).

To confirm that the three-gene ES reflected the previously described cytotoxic-enriched L-TME in the standard-risk subjects of the TARGET cohort, we performed an enrichment analysis using a published immune subpopulation gene set (39). The analysis confirmed that the three-gene score represents a contextual cytotoxic/NK/Th1 expression profile at AML onset, depleted in Q1 and enriched in Q4 ES quartiles (Figure 5e).

Finally, we confirmed an inverse correlation between MRD1 and three-gene ES in this cohort (*p* = 0.014, Figure 5f).

## 4. Discussion

The TME has been extensively described in the solid tumor literature: “hot” T-cell infiltrated tumors have been associated with a favorable prognosis, in contrast to “cold” tumors [23,38].

While the leukemic composition of the AML TME has been extensively investigated [39,40,41], the immune L-TME has been less studied. The recent literature describes how the TME is deeply affected by AML [13], but the correlation between a clear pattern of immune infiltration and prognosis remains controversial [42]. Mazziotta et al. recently correlated a skewed trajectory of CD8 cells toward senescence with a worse prognosis in adult AML patients, analyzed with a multi-dimensional approach onset and relapse paired samples [43].

Our results, while aligning with their findings regarding the onset of pediatric AML leukemia, add data on functional T-cell infiltration and expand the analysis to include CD4^+^, CD19^+^, NK, and NKT cells.

Lasry et al. [44] used single-cell RNA sequencing to profile sorted BM immune cells from 22 pediatric and 20 adult AML patients. They described an 11-gene inflammatory signature (iScore) that included IFN-related genes along with genes related to antigen presentation (HLA genes), growth factor signaling (HGF), and stress signaling oncogenic processes (HSP90AA1), which correlated with a worse prognosis. A key difference from our analysis is that we generated our results by whole RNA-Seq, capturing therefore a broad effect of the interaction between immune and cancer cells, rather than a single-cell immune profile. TARGET data collection, used for validation, is generated by the same whole RNA-seq method. Also we diverge for the selection criteria of the pediatric TARGET validation cohort as their cohort included 336 patients, while we tested a cohort of 833 patients.

In our study, we evaluated the iScore using whole RNA from the TARGET samples we tested (*n* = 833); however, we could not confirm its association with worse prognosis (Supplementary Figure S3(gI–III)). This difference might reflect the diverse analytical frameworks used in the two studies and therefore generate divergent but complementary signatures.

Our results seem to carve a narrower IFN-rich functional signature from this broader inflammatory signature, representing a favorable L-TME. Deep flow cytometry analysis revealed that patients with this type of L-TME benefit from a higher abundance of CD3^+^ T cells enriched with TEMRA, a non-exhausted PD-1 negative phenotype, along with a lower number of infiltrating blasts. Consistently, patients exhibiting an enriched cytotoxic L-TME in the broad TARGET cohort displayed a lower number of blasts at diagnosis, achieved a lower MRD1, and increased long-term OS.

The ES was enriched in TARGET cohort patients assigned to low- and standard-clinical risks, and in general, a favorable high three-gene score (Q4) aligned with good prognostic features, such as AML with *t(8;21)(q22;q22)*, *RUNX1-RUNX1T1*, and absence of *Flt3-ITD* mutations. Accordingly, the unfavorable three-gene-depleted score (Q1) was associated with *Flt3-ITD* mutations and *t(6;9)(p23;q34)*; *DEK-NUP214*, as well as with *NPM1* mutation in the absence of *Flt3-ITD*, *per se* a good prognostic factor.

This finding aligns with a recent very interesting study, by Koedijk et al., where authors perform BM spatial transcriptomics in a cohort of pediatric AML at diagnosis. They describe an M2-like macrophage predominance and immune-depleted samples and that *Flt3-ITD* and/or *NPM1* mutations were associated with low T-cell BM infiltration [45].

It is tempting to assume that certain pediatric AML blast features are more immunogenic than others, triggering a favorable immune expansion at AML onset, making it more prone to control blast invasion, lowering the burden of MRD, and providing better survival.

The chemotherapy regimen used in this study (AAML0531) is a standard treatment for pediatric AML, used worldwide, based on anthracyclines, Aracytin, Etoposide, and an immunosuppressant therapy Gentuzumab Ozogamicin, which is a recombinant humanized anti-CD33 monoclonal antibody. As such, the treatment is mostly myelosuppressive, rather than lympho-suppressive, supporting the hypothesis that a favorable lymphoid composition in the bone marrow could facilitate the blast clearance.

Although our results point more toward an early clearance of blasts, and we did not find any difference in CD4 and CD8 T-stem cell memory enrichment between cohorts, it might also be possible that a pool of leukemic specific T-memory stem cells could better survive chemotherapy within a favorable T cell-rich L-TME, contributing to long-term AML disease control.

Such hypotheses are beyond the scope of this study and may be tested using a dedicated functional study in a larger cohort.

When measured within the clinical risk groups, the three-gene score stratified the OS of standard-risk patients into four quartiles, showing a clear OS advantage for the highest ES quartile (Q4) and a two-fold increased risk of having a reduced OS for patients with the lowest enrichment (Q1). Importantly, the ES was correlated with OS in standard-risk patients, even after removing patients with MRD1 ≥ 0.05% (as per the latest stratification guidelines), proving to be an independent risk score that could offer to “Q1” patients the chance to improve their OS if assigned to an intensified treatment.

A limitation of this study is the small size of our discovery and internal validation cohorts. For this reason, we compromised on statistical stringency during the initial discovery phase by using unadjusted *p*-value thresholds for the three-gene set selection. When we tested the genes set with statistical stability analyses (LOOV and bootstrap), we proved a strong consistency for the three genes in the discovery cohort but a weaker consistency for *TRAT1* gene in the validation cohorts, revealing a potential bias due to individual patient characteristics. Despite this limitation, we decided to maintain the three-gene selection for further analyses, relying on (1) the cohort being representative of three independent recruitment sites (Italy, Qatar, and Pakistan) and (2) the use of NanoString panel at the beginning of the analyses, focusing on subsets of tumor-infiltrating genes and likely favoring a less-sparse L-TME analysis.

Validation experiments using deep phenotype data were also based on a limited number of samples; are to be considered preliminary; and did not allow meaningful correlations between leukemia genotype, phenotype, and T-cell infiltration.

Another limitation of the study is that we used only the AAML1031 edition of TARGET RNA-Seq database for validation, including data collected from patients between years 2011 and 2017. The context of the risk-score analysis does not include, therefore, newer recently discovered genetic markers, as *UBTF* tandem duplications and others that were not available for screening in these years.

In summary, we identified a signature of three-IFN-related genes, representing a non-exhausted T-cell-enriched L-TME at leukemia onset, which correlates with better OS in pediatric AML patients.

A careful flow cytometry evaluation was performed on a limited patient cohort and outlined the following characteristics of this favorable L-TME: (1) a CD3^+^ T-cell expansion with respect to the myeloid and malignant blast compartments (both cell types were not analyzed in detail in our work), (2) a relative increase in TEMRA CD8^+^ T cells, and (3) a less-exhausted phenotype.

Although still preliminary, this discovery, once tested in appropriate clinical trials, has the potential to enhance the stratification of standard-risk patients who currently lack appropriate risk-oriented treatment options. Furthermore, these findings may serve as a valuable roadmap for addressing immune pathways and exploring the potential efficacy of immune-targeted therapies.

## 5. Conclusions

We identified a novel three-gene IFN-related signature that distinguished pediatric AML patients by chemosensitivity and remission outcomes. It stratified patients across all risk groups, including the “standard-risk” group, with high expression linked to a T-cell-enriched microenvironment and longer survival. This signature may enhance risk stratification and guide targeted immunotherapy.

## Data Availability

Data will be made available on reasonable request.

## Supplementary Materials

The following supporting information can be downloaded at https://www.mdpi.com/article/10.3390/cancers18091423/s1, Figure S1. Prognostic HR of IFN signature and T-cell doublets in pediatric AML; Figure S2. T-cell subset gating in BM of pediatric AML patients; Figure S3. BM immune subsets and prognostic three-gene signature in pediatric AML; File S1. Clinical and molecular characteristics of pediatric AML patients, including cohort and subcohort memberships; File S2. The clinical and molecular characteristics of the TARGET pediatric AML RNA-Seq dataset (AAsML1031) were used as the external validation cohort. Patients with available diagnostic BM samples and complete clinical information were included (*n* = 833); File S3. List of antibodies used for extracellular and intracellular staining for flow cytometry analysis; File S4. List of 67 differentially expressed genes (DEGs) identified by NanoString analysis in the discovery cohort. Gene expression profiles were compared between patients with sustained CR (*n* = 6) and those with non-sustained CR (*n* = 19). All DEGs met the significance threshold (*p* < 0.05); File S5. DEGs in the sustained-CR group were identified by mRNA-Seq in the cross-validation subset (*n* = 19). Gene expression profiles were compared between patients with sustained CR (*n* = 5) and those with non-sustained CR (*n* = 14). All DEGs met the significance threshold (*p* < 0.05); File S6. DEGs in the sustained-CR group were identified using mRNA-Seq in the internal validation cohort (*n* = 7). Gene expression profiles were compared between patients with sustained-CR (*n* = 3) and those with non-sustained CR (*n* = 4). All DEGs met the significance threshold (*p* < 0.05); File S7. DEGs in the sustained-CR group were identified by mRNA-Seq of the whole mRNA-Seq cohort (*n* = 26). Gene expression profiles were compared between patients with sustained CR (*n* = 8) and those with non-sustained CR (*n* = 18). All DEGs met the significance threshold (*p* < 0.05); File S8. Functional pathways associated with DEGs between patients with sustained CR (*n* = 8) vs. non-sustained CR (*n* = 18) based on combined mRNA-Seq cohorts (whole mRNA-Seq cohort).

## Abbreviations

The following abbreviations are used in this manuscript:

AML	Acute myeloid leukemia
allo-HSCT	Allogeneic hematopoietic stem cell transplantation
BM	Bone marrow
CR	Complete remission
DEGs	Differentially expressed genes
ES	Enrichment score
FAB	The French–American–British classification
GBP1	Guanylate-binding protein
IFN	Interferon
IPA	Ingenuity pathway analysis
ISGs	Interferon stimulated genes
L-TME	Leukemic bone marrow tumor microenvironment
MRD	minimal residual disease
OS	Overall survival
PARP12	Poly-ADP-ribose-polymerase-12 enzyme
PB	Peripheral blood
RNA-Seq/mRNA-Seq	mRNA-sequencing
ssGSEA	Single-sample gene set enrichment analysis
TARGET	Therapeutically Applicable Research to Generate Effective Treatments
TH1	T helper 1
TME	Tumor microenvironment
TRAT1	T-cell receptor-associated transmembrane adapter
*t*SNE	*t*-distributed stochastic neighbor embedding
WHO	World Health Organization
