# Supplementary material for: A Three-Gene Interferon Signature Predicts Sustained Complete Remission in Pediatric AML Patients
Source: Cancers (Basel). 2026 Apr 29;18(9):1423. doi: 10.3390/cancers18091423 (PMC13162913; doi:10.3390/cancers18091423)
Supplement: Supplementary file 1 [file cancers-18-01423-s001.zip › cancers-4233120-supplementary-figures.pdf]

a

Leave-One-Out Validation (LOOV) test

Gene	Discovery (n=25)	Cross-Validation (n=19)	Internal Validation (n=7)
GBP1	25/25 (100%)	19/19 (100%)	5/7 (71%)
TRAT1	25/25 (100%)	6/19 (32%)	2/7 (29%)
PARP12	25/25 (100%)	19/19 (100%)	7/7 (100%)

Bootstrap resampling test

Gene	Discovery	Cross-Validation	Internal Validation
GBP1	918/1000 (91.8%)	846/1000 (84.6%)	681/1000 (68.1%)
TRAT1	872/1000 (87.2%)	488/1000 (48.8%)	534/1000 (53.4%)
PARP12	837/1000 (83.7%)	964/1000 (96.4%)	812/1000 (81.2%)

b

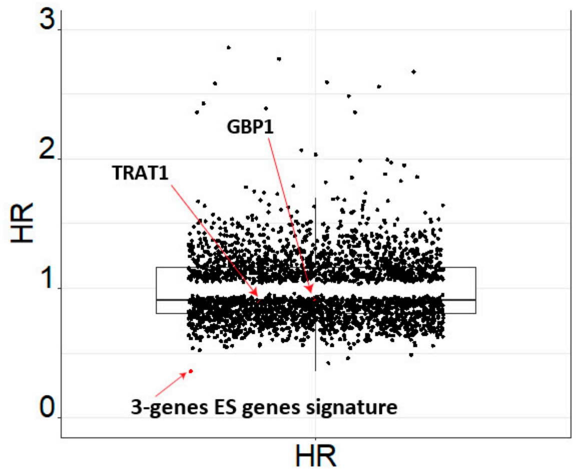

c

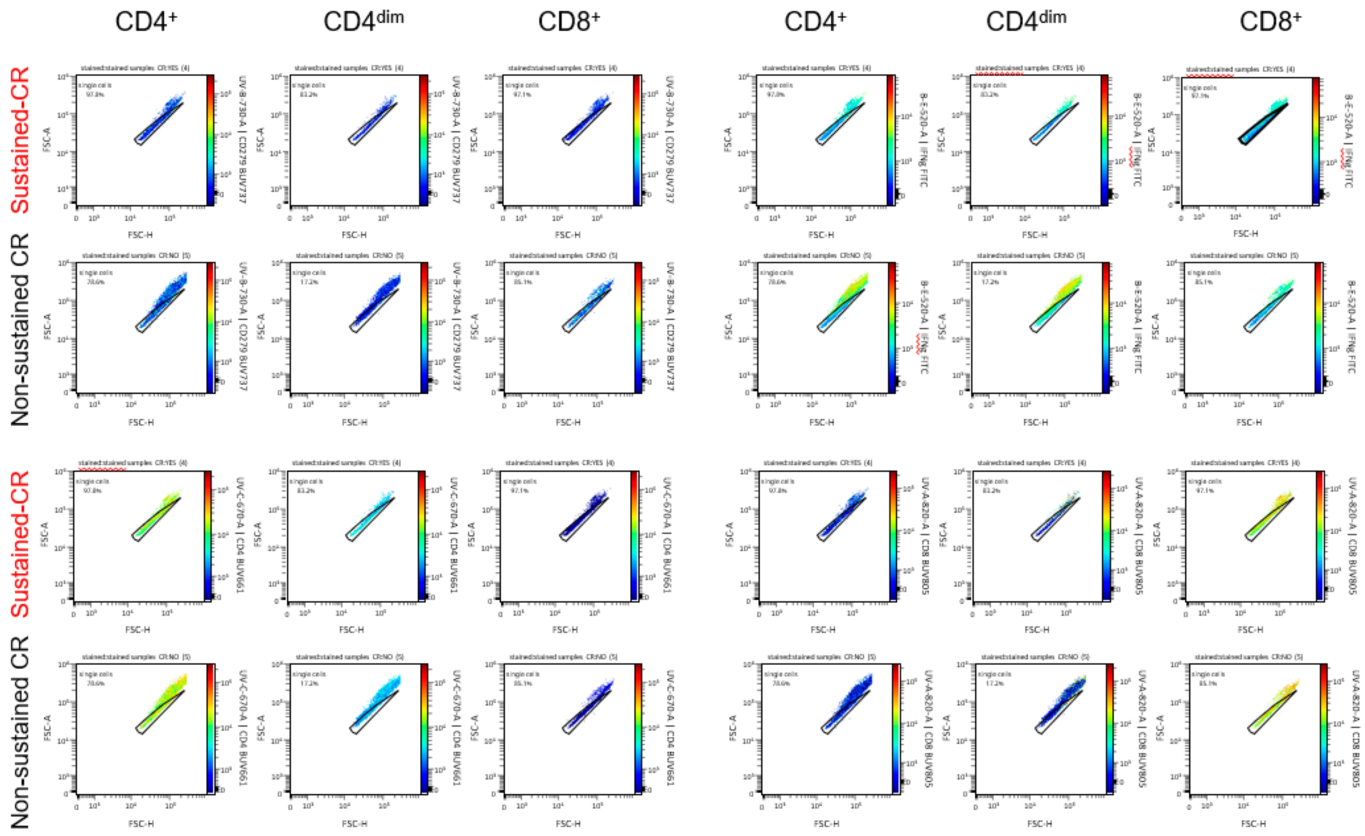

Figure S1. Prognostic HR of IFN signature and T-cell doublets in pediatric AML. (a) Stability and reproducibility of the 3-gene expression signature (GBP1, TRAT1, and PARP12) across multi-cohort validation. Gene signature stability was evaluated using limma ($p < 0.05$) across three cohorts: Discovery (n=25), Cross-Validation (n=19), and Internal Validation (n=7). Upper panel: Leave-One-Out Validation (LOOV). For each cohort, one sample was iteratively withheld, limma differential expression was re-run on the remaining samples, and gene selection frequency was recorded. Results are expressed as the number of iterations in which each gene was selected over the total number of samples per cohort. GBP1 and PARP12 demonstrated consistent selection across all cohorts, while TRAT1 showed reduced stability in the Cross-Validation (6/19, 32%) and Internal Validation (2/7,

29%) cohorts. Lower panel: Bootstrap Resampling (R = 1,000 iterations). Cohort-level robustness was further evaluated by resampling with replacement over 1,000 iterations (seed = 42), with limma differential expression applied at each iteration. Results represent the proportion of Bootstrap iterations in which each gene was identified as differentially expressed. GBP1 showed strong bootstrap stability in the Discovery (91.8%) and Cross-Validation (84.6%) cohorts. PARP12 demonstrated the highest stability in the cross-validation cohort (96.4%). TRAT1 exhibited moderate stability overall, with the lowest performance in the cross-validation cohort (48.8%). (b) Boxplot showing the Cox proportional hazard ratio (HR) for the 3 genes signature and genes with *p*-value < 0.05 (2766 genes) out of the 18000 genes of all RNA-Seq matrices in the standard-risk group of the TARGET Cohort. The 3-genes signature had the lowest HR among all the significant genes. (c) Dot plots representing the cumulative rate of single cells, displayed as FSC-H *vs* FSC-A, across three T-cell populations: $CD8^{+}$ $CD4^{+}$ and $CD8/CD4^{dim}$. In each set of three plots, the Z-axis represents a different antigen measured in patients with sustained-CR (red legend) and non-sustained CR (black legend). The analysis highlighted the increased doublet population within the CD4 ^{dim} gated cells, particularly positive for CD4, CD8, and IFN markers (indicated by the higher orange/red color intensity).

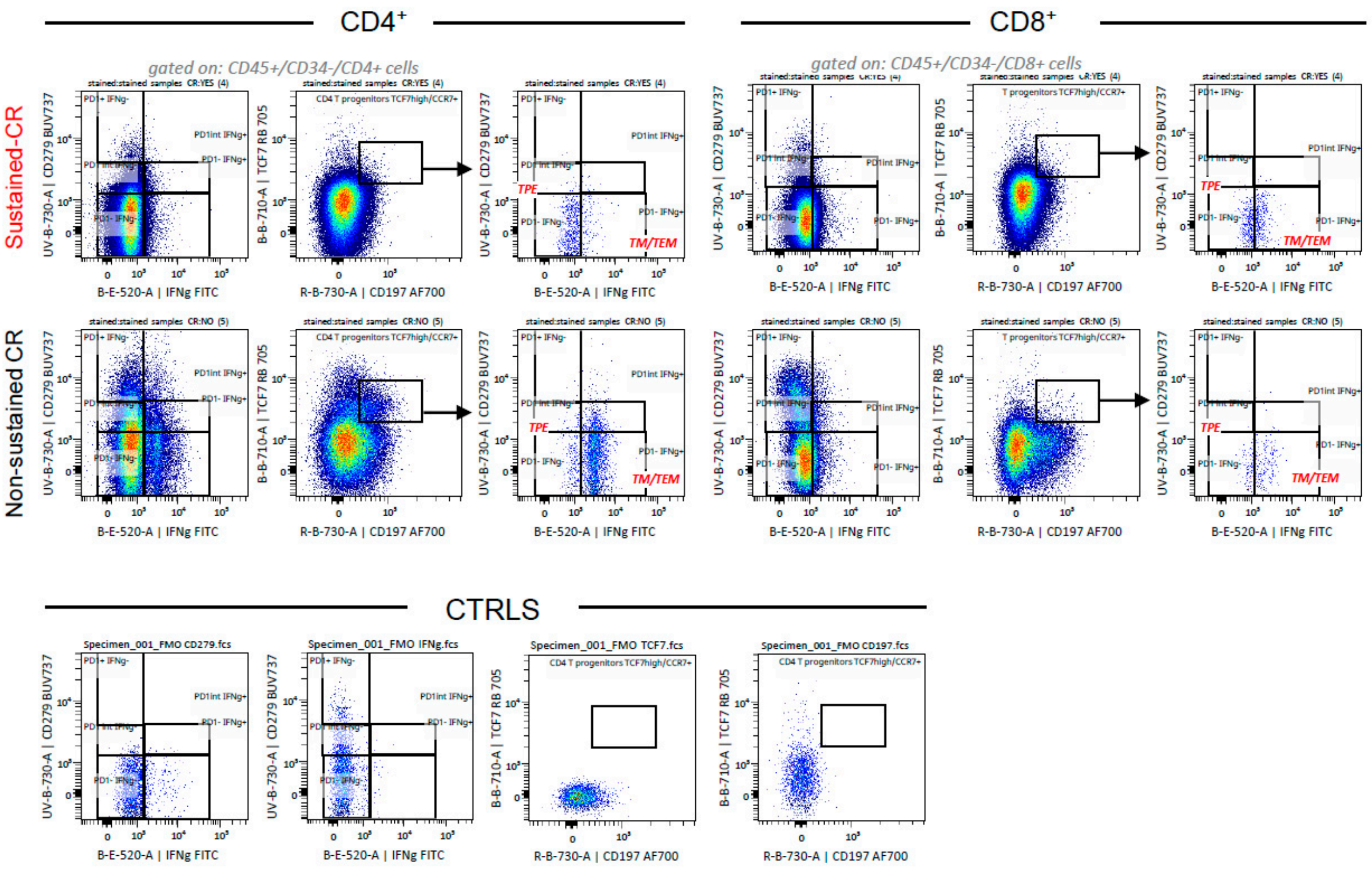

Figure S2. T-Cell subsets gating in BM of pediatric AML patients. Dot plots representing the gating strategy used to analyze T Progenitor exhausted (TPE)and non-exhausted T memory/effector cells (TM/TEM), measured in $CD8^{+}$ and $CD4^{+}$ T-cells populations from patients with sustained (red) and non-sustained CR (black). The individual values are shown in Figure 3. On the left, the PD1 high/int/low gates are shown for the total $CD4^{+}$ and $CD8^{+}$ cell populations. In the middle, the selection of the TCF7 high cells, followed by logical gating measuring PD1 and IFNγ expression in these cells. TPE was defined as IFNγ neg/ PD1 int/low, whereas TM/TEM were identified as PD1 neg/IFNγ high. Cumulative data are shown here, indicating a higher presence of both TPE and TM/TEM cells in the non-sustained CR cohort, although individual differences were not statistically significant (Figure 3). Below are the control gates set up on the FMO samples using the same markers.

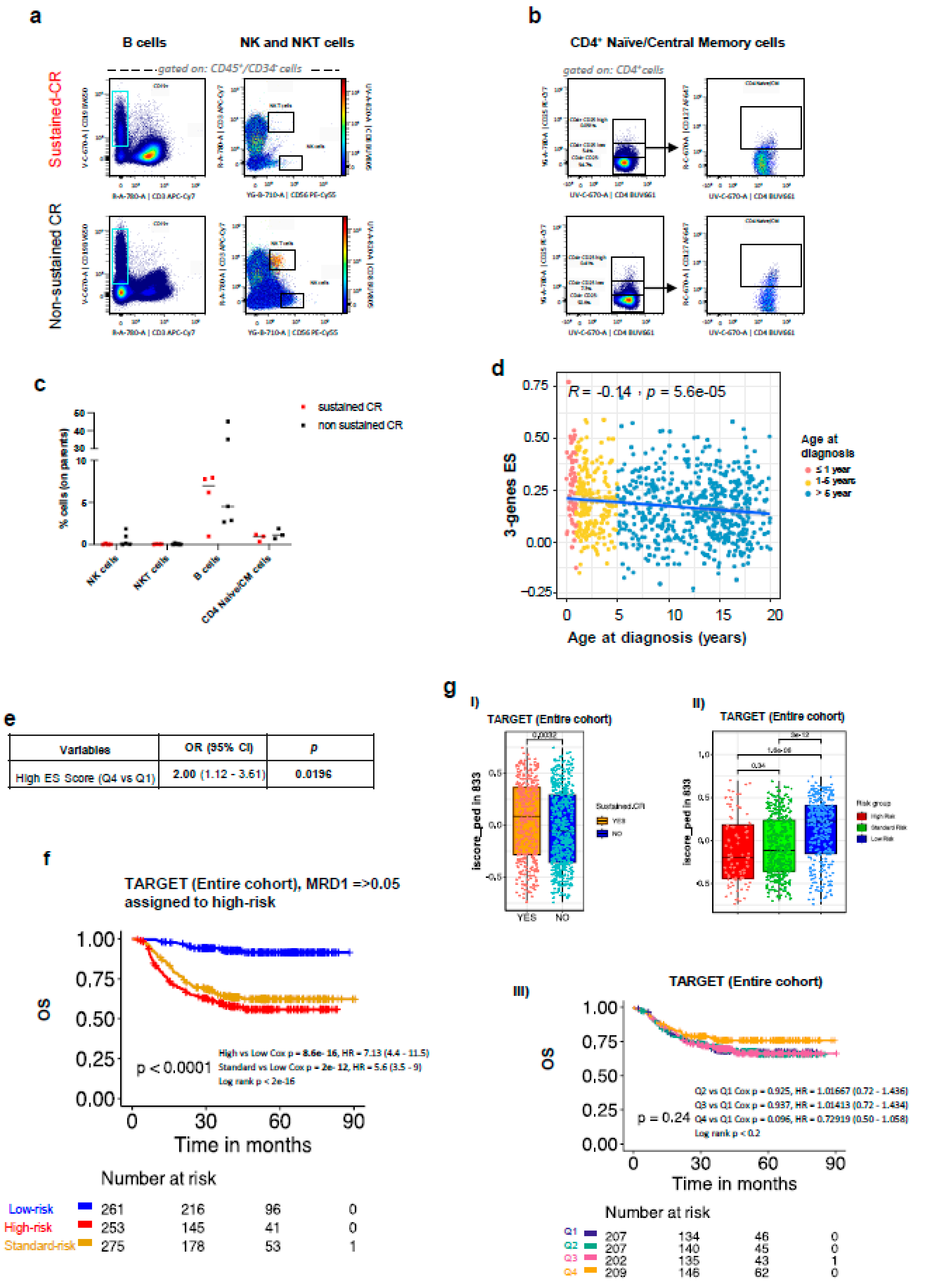

Figure S3. BM immune subsets and prognostic 3-Gene signature in pediatric AML. (a) Dot plots show the cumulative rates of B cells, NK cells, and NKT cells in patients with sustained (red) and

non-sustained CR (black). (b) Logical gating strategy used to identify $CD4^+$ Naïve and Central Memory T cells. (c) Individual values correspond to plots in (a) and (b) for patients with sustained-CR (red dots) and non-sustained CR (black dots). (d) Pearson correlation between the 3-genes ES score and age at diagnosis in the TARGET cohort measured across three age groups: ≤ 1 year, 1-5 years and > 5 years. (e) Logistic regression analysis showing the odds ratios with 95% confidence intervals (CI) of the 3-genes ES quartiles as an explanatory variable for CR in pediatric AML patients with clinical standard-risk in the TARGET cohort. (f) Kaplan-Meier OS curve for clinical-risk groups in the TARGET cohort: low-, standard-, and high-risk groups after re-assignment of all patients with MRD1 ≥ 0.05% in high risk (*n*=789). (g)Analysis of Lasry's pediatric inflammation-associated gene score (iSCORE) in the TARGET AML cohort (*n* = 833): (I) Boxplots displaying the iSCORE defined by Lasry *et al*. (46), based on expression of 12 genes (*COTL1, GSN, HGF, HLA-DQA1, HLA-DQA2, IFI6, IFITM2, RHOG, HLA-DPA1, COMMD3, HSP90AA1*), compared between the sustained-CR and non-sustained CR patients. *P*-value was calculated using Student's *t*-test; the central black line represents the median. (II) Boxplots showing iSCORE distributions across clinical risk groups in the TARGET cohort. *P*-values were calculated using the Student's *t*-test, and the central black line represents the median. (III) Kaplan–Meier OS curve for the TARGET cohort stratified by iSCORE quartiles. The enrichment score was split into four quartiles, with Q1 representing the lowest iSCORE and Q4 being the highest score.
